# Retinal Microvascular Reactivity in Chronic Cigarette Smokers and Non-smokers: An Observational Cross-Sectional Study

**DOI:** 10.3389/fmed.2021.782010

**Published:** 2021-12-20

**Authors:** Huan Xu, Yuan Zong, Jian Yu, Chunhui Jiang, Haohao Zhu, Xinghuai Sun

**Affiliations:** ^1^Department of Ophthalmology and Visual Science, Eye, Ear, Nose and Throat Hospital, Shanghai Medical College of Fudan University, Shanghai, China; ^2^Key Laboratory of Myopia of State Health Ministry and Key Laboratory of Visual Impairment and Restoration of Shanghai, Shanghai, China; ^3^Department of Ophthalmology, Shanghai Fifth People's Hospital, Fudan University, Shanghai, China

**Keywords:** retinal microvasculature, reactivity, optical coherence tomography angiography (OCTA), blood pressure, Valsalva maneuver (VM)

## Abstract

**Purpose:** To evaluate the changes in the retinal microvasculature and its reactivity in chronic cigarette smokers.

**Methods:** Thirty-four male chronic cigarette smokers and 18 male non-smokers were enrolled. Optical coherence tomography angiography was used to measure the perfused retinal vessel densities (PVDs) of the peripapillary and parafoveal areas at baseline and during phase IV of the Valsalva maneuver (VM-IV). Systemic blood pressure and intraocular pressure were also measured.

**Results:** The baseline PVD in the peripapillary area of the smokers was significantly lower than the non-smokers (59.56 ± 2.26% vs. 61.67 ± 3.58%, respectively; *P* = 0.005). However, there was no significant difference in the foveal avascular zone or parafoveal PVD between the two groups. During VM-IV, the peripapillary PVD of the smokers decreased by 1.13 ± 3.50%, which was significantly less than that of the non-smokers (−3.83 ± 4.26%, *P* < 0.05). Similarly, the parafoveal PVD of the smokers decreased by 5.49 ± 9.70%, which was significantly less than the percentage change of the non-smokers (−13.01 ± 8.39%, *P* < 0.05). There was no significant difference in the percentage change in systemic blood pressure parameters between the two groups.

**Conclusion:** The retinal microvasculature and its reactivity were impaired in chronic smokers compared with non-smokers. The extent of impairment differed among different regions of the fundus.

## Introduction

Smoking is a global public health issue, which causes ~7 million deaths each year worldwide (1). Tobacco consumption in China accounts for 40% of global consumption (2). About 300 million individuals were reported to be current smokers in China in 2010, and over half (52.9%) of adult men (288 million) were smokers (3).

Cigarette smoke contains numerous compounds, many of which have toxic and deleterious effect on the vascular system of the human body (4). According to a World Health Organization report, cigarette smoking contributes to 10–30% of all cardiovascular deaths worldwide (5), and several epidemiological surveys have reported that smoking is also strongly associated with ocular vascular diseases, such as retinal ischemia and anterior ischemic optic neuropathy (6). Although the exact pathophysiological process remains unclear, endothelial dysfunction is thought to play a crucial role in the vascular diseases induced by smoking.

The retinal vasculature has an autoregulatory system to maintain constant blood flow and to ensure that the high metabolic needs of the retina are met under various conditions (7). Nesper et al. reported a significant, transient variation of retinal vessel density during the transition from dark adaptation to ambient light (8). Vascular reactivity is a well-known marker of endothelial function. Several studies have demonstrated that the reduction in vascular reactivity is an important and early sign of vascular endothelial dysfunction, which occurs before the appearance of pathological changes (9). Measuring this autoregulatory system involves analyzing the variation in the amplitudes of vascular hemodynamic indices in response to different stimuli, such as flicker stimulation (9–11), elevated blood pressure (BP) (12), inhalation hyperoxia (13), and handgrip test (14). In previous studies in smokers, Halloran et al. reported that the reactivity of retinal arteries was lower in chronic smokers than in non-smokers (15). Garhöfer et al. demonstrated that the response of retinal veins to flicker stimulation also significantly reduced in smokers compared with that in non-smokers (16).

Recently, it has been reported that the capillaries are crucial sites at which blood flow is controlled (17). However, because the techniques available were limited, most of previous studies assessed the reactivity of large vessels, and the reactivity of the capillaries of smokers have not been well-studied. In recent years, the development of optical coherence tomography angiography (OCTA) has allowed clear visualization of retinal capillaries and non-invasive quantification (18). Our previous study demonstrated that OCTA can be used to evaluate the retinal microvascular reactivity to the variations in BP during phase IV of the Valsalva maneuver (VM-IV) (19). In this study, using the same equipment and stimuli, the reactivity of the retinal microvasculature is compared between chronic smokers and non-smokers.

## Materials and Methods

All procedures in this study were reviewed and approved by the Institutional Review Board of the Eye, Ear, Nose, and Throat Hospital of Fudan University in Shanghai, China. The research conformed to the tenets of the Declaration of Helsinki, and the enrolled subjects read and signed a written informed consent form.

### Study Subjects

Healthy subjects who underwent a routine health checkup annually were enrolled in this study. Detailed ophthalmologic examinations were performed in all subjects, including slit-lamp biomicroscopy and a fundus examination. We collected the following basic ophthalmologic data: best-corrected visual acuity (BCVA), intraocular pressure (CT-80A Computerized Tonometer; Topcon, Tokyo, Japan), refractive error (Auto Refractometer AR-610; Nidek Co, Ltd, Tokyo, Japan), and axial length (AL; IOLMaster^®^ Carl Zeiss, Inc., Jena, Germany). The inclusion criteria for the study were: (1) BCVA > 0.8; (2) IOP <21 mmHg; (3) AL of 21–25 mm; (4) a spherical equivalent from −3 to +3 diopters; and (5) no retinopathy or other abnormal ophthalmologic signs. The exclusion criteria were: (1) systemic diseases such as diabetes, hypertension, or other cardiovascular diseases; (2) a history of ocular trauma; (3) inability to correctly perform or complete the VM. The smoking history of the subjects was asked and recorded in detail. Fifty-two healthy male subjects, including 18 non-smokers and 34 chronic smokers, were ultimately enrolled in the study. Non-smokers are defined as subjects who have no smoking history and live in smoke-free environment. Chronic smokers are defined as subjects who have a minimum 10-year smoking history with one or more pack-years. Up to now, the smokers still remain their smoking habits.

### Optical Coherence Tomography Angiography

OCTA scanning of the macular (6.0 × 6.0 mm^2^) and peripapillary regions (4.5 × 4.5 mm^2^) was performed with a spectral-domain OCT system (RTVue-XR Avanti with AngioVue AngioAnalytics, version 2017.100.0.1; Optovue Inc., Fremont, CA, USA), as described in a previous study. The system automatically identifies the parafoveal superficial capillary plexus (signal projected from 3 μm below the internal limiting membrane to the outer boundary of the inner plexiform layer), and generates en face retinal angiograms. The perfused vessel densities (PVDs) in the peripapillary area (a 700-μm-wide elliptical annulus, extending outward from the optic disc boundary) and parafoveal area (an annulus formed by an outer circle with a diameter of 3 mm and an inner circle with a diameter of 1 mm) were automatically calculated with the software. The foveal avascular zone (FAZ) area was also obtained directly from the system. The quality of the OCTA scanning images was determined by the signal strength index (SSI). Finally, the images with an SSI~60 or less were excluded from the analysis. The OCTA system embraced the eye-tracking function to decrease the residual motion artifacts and improve the quality of scanning images.

### Study Protocol

All the subjects were required to refrain from coffee and alcohol consumption for 24 h before the examinations. Before the test, the subjects were asked to rest in a sitting position for 20 min. The baseline retinal PVDs was acquired with the OCTA system, as described above. At the same time, the baseline BP parameters, including the systolic BP (SBP), diastolic BP (DBP), and heart rate (HR), were recorded with a fully automatic BP monitor (HEM-7130; OMRON, Dalian, China). The subjects were then instructed to perform the modified VM by taking a deep breath and forcefully blowing out against the closed glottis while occluding the nose with their fingers. The maximum expiratory pressure had to be sustained for 15 s. The subjects were then required to open their glottis and breath normally. As previously described, phase III of VM started with the release of pressure and lasts for 1–2 s, with a transient fall in BP, and was followed by an overshoot of BP in phase IV, which last about 10–20 s (20). To investigate the retinal microvascular response to the elevation in BP induced in VM-IV, OCTA images were obtained 5 s after VM release. Because it took 5–7 s to pressurize the cuff, the examiner pressed the “start” button of the BP monitor when the VM strain was released. Therefore, the BP parameters and retinal angiogram images were acquired at almost the same time. After resting for 1 h, the subjects performed a second VM, and IOP was measured 5 s after VM release.

### Statistical Analysis

Spherical equivalence (SE) was calculated as the algebraic sum of the spherical value plus one half of the cylindrical value. The mean arterial pressure (MAP) and ocular perfusion pressure (OPP) were calculated with the following formulae:

MAP = 1/3 (2 × DBP + SBP)

OPP = 2/3 MAP–IOP

The response of the retinal PVDs to VM-IV was expressed as “Δ,” which was equal to the percentage change relative to the baseline level. The changes in the systemic parameters were calculated using similar methods. To investigate the effects of VM on the various parameters, paired *t*-tests were used to compare the differences in the parameters between baseline and during VM-IV in the same group. The one-sample Kolmogorov-Smirnov test and Levine's test were used to test the normality of distributions and the equality of variance, respectively, of all datasets. According to the results, the SE value, ΔFAZ, and ΔDBP were compared between the two groups with Mann-Whitney *U*-tests. All other baseline parameters were compared with two-tailed independent-samples *t*-tests. The differences of changes of the other parameters between the two groups (non-smokers vs. chronic smokers) were compared with the general linear model multivariate analysis, which could adjust for the potential confounders (age, AL, and BCVA). A linear regression model was used to analyze the correlation between pack-years and the response of the retinal PVDs to VM-IV in parafoveal and peripapillary regions. Data are presented as means ± standard deviations and statistical significance was set at *P* < 0.05. All statistical analyses were performed with SPSS software (version 20.0; SPSS, Inc., Chicago, IL, USA).

## Results

### Subject Characteristics

The right eyes of 34 male chronic smokers and 18 male non-smokers were enrolled in the study. For chronic smokers, the mean smoking history last for 23.53 ± 7.95 years. The average amount of smoking cigarettes was 26.51 ± 12.15 pack-years. Table 1 shows all the subjects' demographic and ocular characteristics. There was no significant difference between the two groups in terms of their age or basic ocular indices, including BCVA, SE, AL, and IOP (all *P* > 0.05; Table 1).

**Table 1:** Subjects characteristics.

	**Non-smokers**	**Smokers**	***P***
Age (y)	43.88 ± 11.42	48.23 ± 9.94	0.165
BCVA (LogMAR) (Snellen)	0.01 ± 0.17 (20/20)	0.02 ± 0.11 (20/21)	0.823
SE (diopters)	−0.66 ± 1.49	−0.16 ± 0.92	0.093
AL (mm)	24.12 ± 0.91	23.65 ± 1.07	0.061
IOP (mmHg)	13.11 ± 3.23	12.85 ± 2.05	0.783

*Data are presented as mean ± standard deviation*.

*BCVA, best-corrected visual acuity; SE, spherical equivalent; AL, axial length; IOP, intraocular pressure*.

*P, The SE values between the two group was compared with Mann-Whitney U-tests. All other parameters were compared with two-tailed independent sample t-tests*.

### Baseline BP and Retinal PVDs

Compared with the non-smokers, the smokers had similar baseline values in several systemic and ocular parameters, including SBP, DBP, HR, MAP, and OPP (all *P* > 0.05; Table 2). The peripapillary PVD was significantly lower in the smokers than in the non-smokers (59.56 ± 2.26% vs. 61.67 ± 3.58%, respectively; *P* = 0.005; Table 3). However, there was no significant difference in FAZ or parafoveal PVD between the smokers and non-smokers (both P > 0.05; Table 3).

**Table 2:** Systemic parameter and other ocular findings before and after VM-IV in non-smokers and smokers.

	**Baseline**			**VM-IV response (%)**		
	**Non-smokers**	**Smokers**	***P* ^a^**	**Non-smokers**	**Smokers**	***P* ^b^**
SBP (mmHg)	119.33 ± 15.27	122.88 ± 13.39	0.390	7.47 ± 8.43^*^	6.08 ± 9.53^†^	0.606
DBP (mmHg)	76.29 ± 13.32	80.12 ± 9.09	0.282	6.11 ± 14.03^*^	4.48 ± 10.28^†^	0.635
MAP (mmHg)	90.93 ± 12.72	94.37 ± 10.01	0.288	6.50 ± 10.45^*^	5.13 ± 8.56^††^	0.613
IOP (mmHg)	13.11 ± 3.23	12.85 ± 2.05	0.783	1.24 ± 7.89	0.48 ± 6.38	0.707
OPP (mmHg)	47.56 ± 7.89	45.04 ± 10.20	0.366	8.34 ± 13.25^*^	6.65 ± 11.26^††^	0.630
HR (bpm)	72.67 ± 9.63	72.70 ± 9.00	0.991	−1.52 ± 2.87	−2.80 ± 8.51	0.437

*VM-IV, phase IV of Valsalva maneuver; SBP, systolic blood pressure; DSP, diastolic blood pressure; MAP, mean arterial pressure; IOP, intraocular pressure; OPP, ocular perfusion pressure; HR, heart rate*.

* *P < 0.05, Changes in parameters of non-smokers during VM-IV was tested with paired t-tests*.

† *P < 0.05*,

†† *P < 0.01, Changes in parameters of smokers during VM-IV was tested with paired t tests*.

P^a^ *, Comparison of baseline parameters between non-smokers and smokers, tested with two-tailed independent sample t-tests*.

P^b^ *, Comparison of percentage changes in parameters induced by VM-IV between non-smokers and smokers, tested with two-tailed independent sample t-tests and Mann–Whitney U-tests (DBP)*.

**Table 3:** FAZ area and retinal PVDs before and after VM-IV in non-smokers and smokers.

	**Baseline**			**VM-IV response (%)**		
	**Non-smokers**	**Smokers**	***P* ^a^**	**Non-smokers**	**Smokers**	***P* ^b^**
FAZ area (mm^2^)	0.33 ± 0.17	0.38 ± 0.12	0.232	9.23 ± 16.51^*^	8.74 ± 18.77^†^	0.413
Parafoveal PVD (%)	51.53 ± 0.98	51.95 ± 0.71	0.571	−13.01 ± 8.39^**^	−5.49 ± 9.70^††^	0.017^#^
Peripapillary PVD (%)	61.67 ± 3.58	59.56 ± 2.26	0.005^‡^	−3.83 ± 4.26*	−1.13 ± 3.50	0.036^#^

*VM-IV, phase IV of Valsalva maneuver; FAZ, foveal avascular zone; PVD, perfused vessel density*.

* *P < 0.05*,

** *P < 0.01, Changes in parameters of non-smokers during VM-IV was tested with paired t-tests*.

† *P < 0.05*,

†† *P < 0.01, Changes in parameters of smokers during VM-IV was tested with paired t-tests*.

P^a^ *< 0.05, Comparison of baseline parameters between non-smokers and smokers, tested with two-tailed independent-samples t-tests*.

^#^P^b^ *< 0.05, ^##^P^b^ < 0.01, Comparison of percentage changes in parameters during VM-IV between non-smokers and smokers, tested with two-tailed independent-samples t-tests and Mann–Whitney U-tests (FAZ area)*.

### Response to VM-IV

Compared with the baseline levels, there were significant increases in SBP, DBP, MAP, and OPP during VM-IV in the smokers and non-smokers (all *P* < 0.05; Table 2). However, IOP and HR did not significantly change in either group (both *P* > 0.05; Table 2). There was no significant difference between the two groups in the percentage change in SBP, DBP, MAP, OPP, IOP, or HR (Table 2).

**Figure 1:**
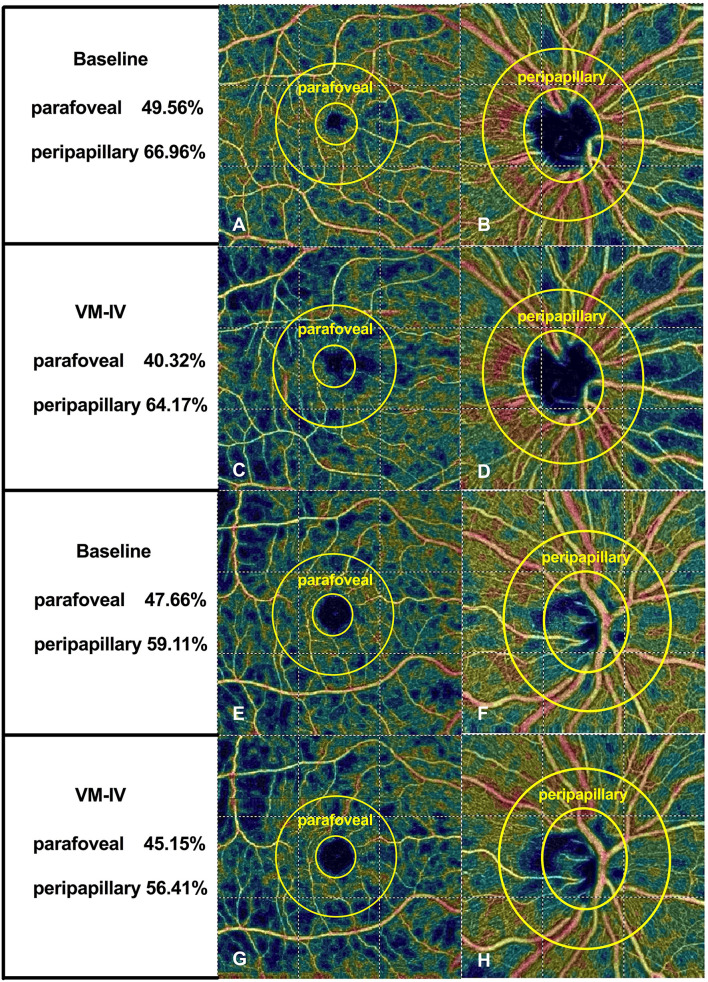
Typical optical coherence tomography angiographic scanning images of non-smokers **(A–D)** and smokers **(E–H)** at baseline and during phase IV of the Valsalva maneuver.

During VM-IV, the peripapillary PVD and parafoveal PVD of the nonsmokers decreased significantly by 3.83 ± 4.26% and 13.01 ± 8.39%, respectively (both *P* < 0.05; Figure 1, Table 3). However, in the smokers, the peripapillary PVD remained unchanged (−1.13 ± 3.50%, *P* > 0.05) and only the parafoveal PVD significantly decreased (−5.49 ± 9.70%, *P* < 0.05; Figure 1, Table 3).

The percentage reductions in retinal PVDs (ΔPVD%) were compared between the two groups with the general linear model multivariate analysis. After adjusting for the potential confounders (age, AL and BCVA), the peripapillary ΔPVD% was significantly greater in the non-smokers than in the smokers (P = 0.036; Figure 1, Table 3). And the parafoveal ΔPVD% were also significantly greater in the non-smokers than in the smokers (*P* = 0.017; Figure 1, Table 3). Meanwhile, age was a significant factor that affected the comparison of parafoveal ΔPVD% between the two groups (*P* = 0.028). The FAZ area increased significantly in both the non-smokers and smokers (9.23 ± 16.51% vs. 8.74 ± 18.77%, respectively; both *P* < 0.05; Table 3), and ΔFAZ% was similar in both groups (*P* = 0.413; Table 3).

For both smokers and non-smokers, the increase in BP during VM-IV induced a greater reduction in retinal PVD in the parafoveal region than in the peripapillary region (both *P* < 0.05; Table 4).

**Table 4:** Comparisons of retinal microvascular reactivity to VM-IV between parafoveal region and peripapillary region.

	**ΔParafoveal PVD (%)**	**ΔPeripapillary PVD (%)**	***P***
Non-smokers	−13.01 ± 8.39	−3.83 ± 4.26	<0.001^**^
Smokers	−5.49 ± 9.70	−1.13 ± 3.50	0.005^**^

*VM-IV, phase IV of Valsalva maneuver; PVD, perfused vessel density*.

*Δ, equal to a percentage change during VM-IV relative to the baseline levels*.

** *P < 0.01, tested with paired t-tests*.

**Figure 2:**
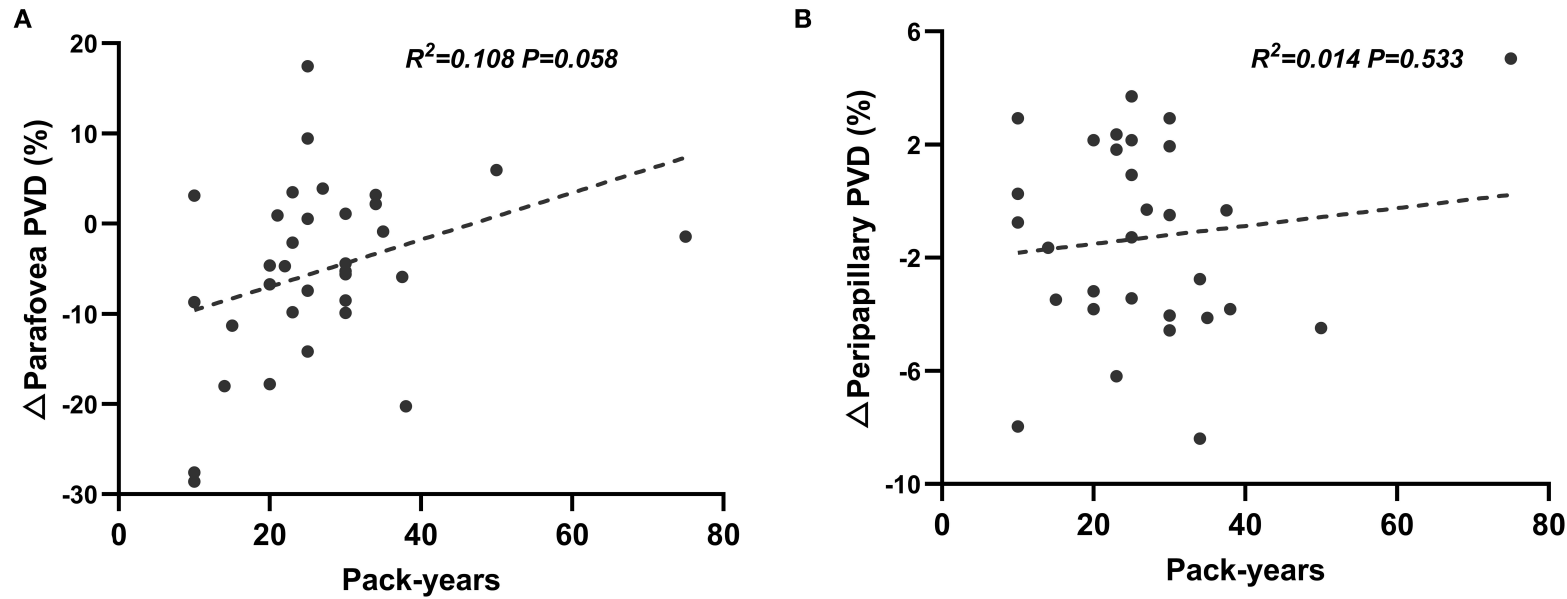
Correlation between pack-years and percentage changes of perfused vessel density induced by Valsalva maneuver in parafoveal **(A)** and peripapillary **(B)** regions.

Linear regression analyses revealed that there was no significant correlation between pack-years and retinal microvascular response induced by VM-IV in either peripapillary region (β = 0.444, *P* = 0.553) or parafoveal region (β = 0.415, *P* = 0.058; Figure 2).

## Discussion

This study used OCTA to assess the potential damage of the retinal microvasculature and its reactivity in smokers. Compared with the non-smokers, the retinal microvasculature and its reactivity were impaired in the smokers, and the extent of impairment differed in different regions of the fundus.

The retinal PVD in the peripapillary area was lower in smokers than in non-smokers. A previous study found that nicotine, a major ingredient of tobacco, has direct toxic effect on the endothelium and smooth muscle cells (SMCs), leading to the loss of capillary vessels (21, 22). Several studies have also indicated that smoking increases the release of vasoconstrictive agents, such as endothelin (23). Consequently, vasoconstriction or the loss of retinal vessels may contribute to the reduced PVD detected in smokers.

Interestingly, no significant difference was found in the baseline parafoveal PVD between smokers and non-smokers. Although the detailed mechanism is still unclear, some inferences can be drawn from this phenomenon. First, four major retinal arteries and veins are located in the peripapillary region, whereas the parafoveal region mainly contains capillaries. In the retina, the larger vessels, such as arteries and arterioles, are encircled by vascular smooth muscle cells (VSMCs), whereas the capillaries are not fully covered by pericytes and their processes (24). Therefore, the major cells controlling the vascular tone differ in the parafoveal and peripapillary regions. Wang et al. found that chronic nicotine exposure elevated the resting intracellular calcium ion concentration and upregulated the expression of the canonical transient receptor potential channels in cultured VSMCs, which are responsible for the increased vascular tone (25). Thus, this may cause a difference in the extent of vessel contraction in different regions of the retina, explaining the different changes in vessel density in smokers.

The evaluation of retinal vascular reactivity is a sensitive diagnostic method because it reflects endothelial function; and retinal vascular reactivity is often impaired in the early stage of diseases, even before vascular structural damage is apparent (26). Using handgrip test, Sousa et al. found an early impairment of the retinal vascular reactivity in patients with type 1 diabetes without clinical diabetic retinopathy (27). VM is a simple, effective, and non-invasive method that is widely used as a diagnostic procedure in several disciplines (28, 29). The VM is divided into four physiological phases according to the BP response. The “overshoot” of BP occurs during VM-IV and lasts for ~10–20 s as a result of the sustained vasoconstriction commencing in phase II. Therefore, VM is also used as a standard stimulus to evaluate the regulatory ability of the cardiac and cerebral vascular systems during variations in BP (29). The present study was designed to investigate the retinal microvascular reactivity to the elevation in BP during VM-IV.

Our study revealed that the retinal microvascular response to VM-IV in the peripapillary and parafoveal areas was significantly lower in smokers than in non-smokers. This result is consistent with previous reports. O'Halloran et al. reported that the magnitude of vasoconstriction in the retinal arteriole response to hyperoxia reduced in smokers compared to non-smokers (30). Similarly, Garhöfer et al. demonstrated that the hemodynamic response of retinal veins induced by flicker stimulation reduced in chronic smokers (16). However, the exact mechanism underlying impaired vascular reactivity has not been fully clarified. Our results show that VM-IV induced increases in BP and the ocular perfusion pressure. Under normal conditions, the retinal hemodynamic response to increasing OPP is caused by an increase in vascular resistance (12). This behavior is called the “myogenic response,” and is controlled by VSMCs (7). The active contraction of VSMCs is responsible for counteracting the increased transmural pressure. Neymar et al. provided experimental evidence that chronic smoking induces hypoxemia and hypercapnia (31), and a recent study demonstrated that hypercapnia impairs the myogenic regulation of retinal vessels in response to changes in BP (32). Exposure to nicotine also induced the phenotypic transformation of SMCs from the contractile type to a synthetic-like type, which might attenuate the sensitivity of the myogenic response (22). These lines of evidence may partly explain the present findings.

The result showed that age was a significant factor affecting the comparison of parafoveal ΔPVD% between the two groups. This result could be explained by the previous study. Yu et al. demonstrated that age was negatively associated with baseline macular vessel density (33). Additionally, our group reported that age also has negative correlation with the change in parafoveal vessel density during VM-IV (19). In the present study, the average age of smokers was slightly higher than the non-smokers, although there was no statistical difference between the two groups. Thus, age become a significant confounder affecting the difference in parafoveal ΔPVD% in the multivariate analysis.

For smokers, the borderline level of statistical significance (*P* = 0.058) was obtained from linear regression analysis of the correlation between pack-years and retinal parafoveal ΔPVDs. This result might be due to the limited number of enrolled subjects. However, to some extent, it could reveal that the increasing cigarette consumption has strong tendency to be negatively correlated with the retinal microvascular reactivity. Future research with larger sample size will be conducted to validate this finding.

For both smokers and non-smokers, the increase in BP induced a greater reduction in retinal PVD in the parafoveal region than in the peripapillary region. This result is consistent with previous studies, which demonstrated differential vascular reactivity in different regions. Assam et al. demonstrated that the changes in the hemodynamic parameters are smaller in the nasal region than in the temporal region, which contains the macular area (34). In previous studies using OCTA, the retinal microvascular response to VM is greater in the parafoveal region than in the peripapillary region (19, 35). All of these results indicate that the greater vascular autoregulatory capacity of the macular vessels may better adapt them to various conditions or stimuli, in order to maintain the relatively constant blood flow required to supply the highly metabolic macula tissue.

There are several drawbacks in this study. Firstly, only a small group of Chinese males was included in this study, so the findings may not be generalizable to other populations. Further studies that include more subjects of different ages, sexes, and backgrounds are required. The exact mechanisms of the damage caused by smoking and the potential benefits of quitting smoking will be evaluated in the further. Secondly, this study was conducted in 2017, when the OCTA image acquisition system has not been updated. Thus, in parafoveal region, only the superficial capillary plexus could be identified and the perfused vessel density in this layer could be automatically quantified. In the future, we will compare the difference of retinal vascular reactivity in different capillary layer using the updated OCTA system. Lastly, due to the limited space between the subject's head and the instrument, we chose a modified VM instead of the classical VM developed by Levin (36). A modified VM is done by forcefully expiring against a closed glottis. As the pressure of forceful expiration could not be measured numerically, the repeatability and reproducibility of the modified VM might be lower than that of the classical method.

In conclusion, the results presented here, together with previous findings, demonstrate the potential damage to the retinal vascular system caused by smoking, although the degree of impairment may differ in different regions of the retina. Further studies in this field are required, and particular attention should be paid to potential variations in the sizes and locations of the affected vessels.

## Data Availability Statement

The raw data supporting the conclusions of this article will be made available by the authors, without undue reservation.

## Ethics Statement

The studies involving human participants were reviewed and approved by Institutional Review Board of the Eye, Ear, Nose, and Throat Hospital of Fudan University in Shanghai, China. The patients/participants provided their written informed consent to participate in this study.

## Author Contributions

HX: conception and design. CJ, XS, and HZ: administrative support. CJ and HZ: provision of study materials or patients. HX, YZ, and JY: collection and assembly of data. HX and YZ: data analysis and interpretation. All authors wrote the manuscript and final approval of manuscript.

## Funding

This study was supported, in part, by research grants from the National Natural Science Foundation of China (82070980), the National Key Research & Development Plan (2017YFC0108200), the Shanghai Committee of Science and Technology (19441900900), and Jiangsu Province Key Research & Development Program (BE2018667).

## Conflict of Interest

The authors declare that the research was conducted in the absence of any commercial or financial relationships that could be construed as a potential conflict of interest.

## Publisher's Note

All claims expressed in this article are solely those of the authors and do not necessarily represent those of their affiliated organizations, or those of the publisher, the editors and the reviewers. Any product that may be evaluated in this article, or claim that may be made by its manufacturer, is not guaranteed or endorsed by the publisher.
